# Incidence of the Beet Leafhopper-Transmitted Virescence Agent Phytoplasma in local Populations of the Beet Leafhopper, *Circulifer tenellus*, in Washington State

**DOI:** 10.1673/031.010.1801

**Published:** 2010-03-15

**Authors:** Joseph E. Munyaneza, James M. Crosslin, Jeffrey E. Upton, Jeremy L. Buchman

**Affiliations:** ^1^ USDA-ARS, Yakima Agricultural Research Laboratory, Wapato, WA 98951; ^2^ USDA-ARS, Vegetable and Forage Crops Research Unit, Prosser, WA 99350

**Keywords:** potato, purple top disease, beet leafhopper, phytoplasma infectivity

## Abstract

Phytoplasma diseases are increasingly becoming important in vegetable crops in the Pacific Northwest. Recently, growers in the Columbia Basin and Yakima Valley experienced serious outbreaks of potato purple top disease that caused significant yield loss and a reduction in tuber processing quality. It was determined that the beet leafhopper-transmitted virescence agent (BLTVA) phytoplasma was the causal agent of the disease in the area and that this pathogen was transmitted by the beet leafhopper, *Circulifer tenellus* Baker (Hemiptera: Cicadellidae). To provide the most effective management of phytoplasmas, timing of insecticide applications targeted against insects vectoring these pathogens should be correlated with both insect abundance and infectivity. Beet leafhoppers were collected from a potato field and nearby weeds in Washington throughout the 2005, 2006, and 2007 growing seasons and tested for BLTVA by PCR to determine the incidence of this phytoplasma in the insects. In addition, overwintering beet leafhoppers were collected throughout Columbia Basin and Yakima Valley and tested for BLTVA to investigate if these insects might constitute a source of inoculum for this phytoplasma from one season to the next. Results showed that 29.6% of overwintering leafhoppers collected near potato fields carried the phytoplasma. BLTVA-infected leafhoppers were also found in both potatoes and nearby weedy habitats throughout the growing season. PCR testing indicated that a large proportion of beet leafhoppers invading potatoes were infected with the phytoplasma, with an average of 20.8, 34.8, and 9.2% in 2005, 2006, and 2007, respectively. Similarly, BLTVA infection rate in leafhoppers collected from weeds in the vicinity of potatoes averaged 28.3, 24.5, and 5.6% in 2005, 2006, and 2007, respectively. Information from this study will help develop action thresholds for beet leafhopper control to reduce incidence of purple top disease in potatoes.

## Introduction

In recent years, diseases caused by phytoplasmas have become increasingly important in vegetable crops of the Pacific Northwest. Since 2002, potato growers in the Columbia Basin of Washington and Oregon and Yakima Valley have experienced serious outbreaks of potato purple top disease that caused significant yield losses and a reduction in tuber processing quality (Munyaneza 2005; Munyaneza et al. 2007). In addition, similar disease outbreaks were observed in several vegetable crops in the same area, including dry beans, carrots, and radish grown for seed (Lee et al. 2004a, 2006; Munyaneza, unpublished data). It has been determined that the beet leafhopper-transmitted virescence agent (BLTVA) phytoplasma (also known as the Columbia Basin potato purple top phytoplasma and belonging to clover proliferation phytoplasma group [16SrVI], subgroup 16SrVI-A) is the causal agent of the disease in the Columbia Basin and Yakima Valley. This pathogen is transmitted by the beet leafhopper, *Circulifer tenellus* Baker (Hemiptera: Cicadellidae) (Lee et al. 2004b; Crosslin et al. 2005; Munyaneza and Upton 2005; Munyaneza et al. 2006a, 2007). Despite the increasing importance of the problem, little is known about the BLTVA infectivity of beet leafhoppers invading potatoes and other vegetable crops and timing of plant infection. To provide the most effective management of phytoplasma diseases, insecticide applications targeted against insects vectoring these pathogens should be timed to correlate with both insect vector abundance and infectivity (Weintraub and Beanland 2006). This pest management strategy has widely been used in the central states of the United States to manage aster yellows disease in several crops, including potatoes. The aster yellows index, which is based on numbers of the aster leafhopper, *Macrosteles quadrilineatus*, combined with phytoplasma infectivity, has been developed to initiate control measures before the pathogen is widely transmitted to susceptible crops (Mahr et al. 1993; Foster and Flood 1995; Capinera 2001). The main objective of the present study was to determine the incidence of BLTVA phytoplasma in beet leafhoppers collected from potatoes and nearby weeds throughout the growing season in Washington. In addition, overwintering beet leafhoppers were collected throughout the Columbia Basin and Yakima Valley and tested for BLTVA to investigate if overwintering insects might constitute a source of inoculum for this phytoplasma from one year to the next.

## Materials and Methods

During the 2005, 2006, and 2007 growing seasons, beet leafhoppers were collected from an 8-acre untreated potato field planted at the USDA-ARS Research Farm near Yakima, WA. The weeds in the vicinity of the potato field were also sampled for the beet leafhopper. Weeds included grasses (*Bromus* spp.), mustards (*Brassica* spp., *Sysymbrium altissimum* L. [Capparales: Brassicaceae]), kochia (*Basia scoparia* (L.) Scott [Caryophyllales: Chenopodiaceae]), filaree (*Erodium cicutarium* (L.) L'Her [Geraniales: Geraniaceae]), Russian thistle (*Salsola tragus* L. [Caryophyllales: Chenopodiaceae]), rabbitbrush (*Chrysothamnus* spp.), sagebrush (*Artemisia* spp.), prickly lettuce (*Lactuca serriola* L. [Asterales: Asteraceae]), hoary cress (*Cardaria draba* (L.) Desv. [Capparales: Brassicaceae]), and pigweed (*Amaranthus* spp.). Heavy-duty sweep nets (BioQuip Products, Inc., www.bioquip.com), each with a 30 cm diameter net hoop, were used during the leafhopper sampling. Four 100-sweep samples were taken weekly between May and late August in both weeds and potatoes. Insects were collected in plastic bags, placed in coolers, and brought to the laboratory at the USDA-ARS in Wapato, WA.

A survey was conducted during the winter and early spring of 2005 throughout the Yakima Valley and the Columbia Basin of Washington and Oregon, to determine the incidence of BLTVA in overwintering beet leafhoppers. Sampling sites included Alderdale, Ephrata, Mattawa, Moses Lake, Moxee, Othello, Paterson, Quincy, and Warden in Washington, and Boardman and Hermiston/Umatilla area in Oregon. During this survey, beet leafhoppers were collected on overwintering weeds (mainly composed of mustards) in several locations near commercial potato fields. Because weeds were too small to be swept at this time of the year, a D-VAC suction device (Rincon-Vitova, www.rinconvitova.com) was used to collect these overwintering beet leafhoppers. D-VAC samples were collected and processed as above.

Beet leafhoppers were sorted from the sweep net and D-VAC samples. The beet leafhoppers were then preserved in 70% alcohol until tested for BLTVA by PCR. Insect testing was performed at the USDA-ARS in Prosser, WA. A sample of 15 beet leafhoppers was tested for each sampling date at the USDA-ARS Research Farm over the three years. During the leafhopper overwintering survey, a total of 189 beet leafhoppers were collected from January to March and a sample of 98 insects was selected and tested for BLTVA by PCR. The nucleic acid extractions from individual insects were conducted as described by Crosslin et al. (2005). The first-round PCR reactions were performed using primer pair Pl and P7 and nested reactions used primer pair fU5 and BLTVA-int as described previously (Crosslin et al. 2005). Insects were considered positive for phytoplasma if the expected amplification product of ≈1.2 kbp was visible after agarose gel electrophoresis.

Data on the incidence of BLTVA phytoplasma in beet leafhoppers over the three years were statistically analyzed with multiway categorical methods, testing for association between three variables: presence or absence of BLTVA in insects, collection date, and host plant (weeds or potatoes). Because BLTVA infection status in beet leafhoppers can be considered a response variable, and the remaining two variables potential explanatory variables, a logit model was developed. The resulting model is analogous to a regression model, except that response and explanatory variables are categorical. The analyses were performed using PROC CATMOD (SAS Institute 2003). The level of significance was set at *p* = 0.05.

## Results

**Figure 1:**
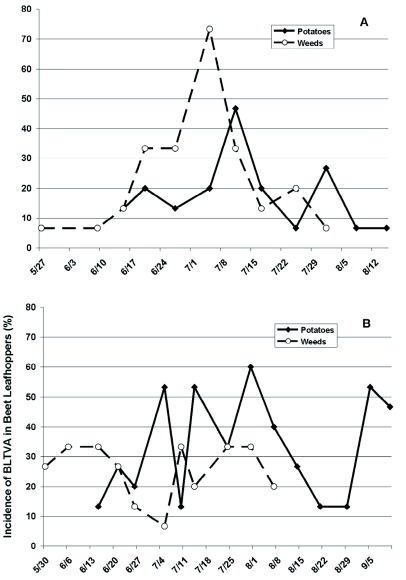
Incidence of BLTVA phytoplasma in beet leafhoppers collected from potatoes and nearby weeds at the USDA-ARS Research Farm near Yakima, WA in 2005 (A), 2006 (B), and 2007 (C).High quality figures are available online.

BLTVA-infected beet leafhoppers were found in both potatoes and nearby weeds throughout the growing season during the three years of study at the USDA-ARS Research Farm (Fig. 1). Phytoplasma testing results indicated that BLTVA incidence in beet leafhoppers in 2005 averaged 20.8 and 28.3% of leafhoppers collected from potatoes and weeds, respectively (Fig. 1A).

**continued:**
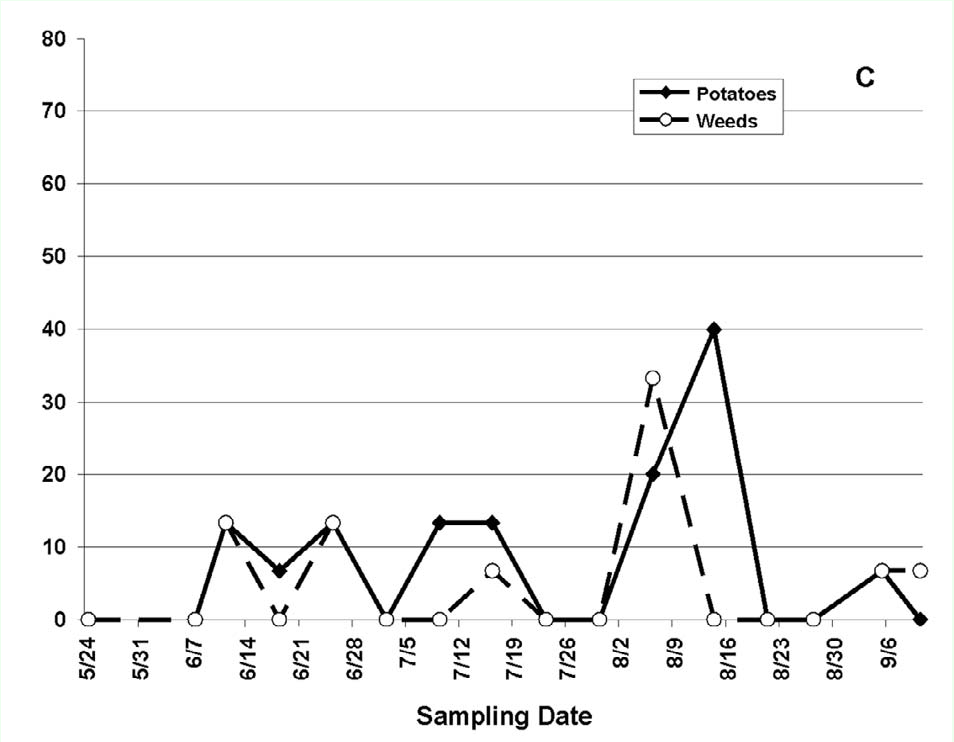

Results of the loglinear model assessing the combined effects of sampling date and host plant showed significant sampling date effects (*p* = 0.0190) but non-significant host plants effects (*p* = 0.1622). The model includes only those dates for which both weeds and potatoes were sampled (Fig. 1). The likelihood ratio was marginally significant (*p* = 0.055), which is evidence that the main effects model fit the data relatively poorly.

In 2006, BLTVA incidence in insects averaged 34.8 and 24.5% of leafhoppers found in potatoes and weeds, respectively (Fig. 1B). Loglinear model analysis of the 2006 data (Fig. 1B) showed that there were no significant effects of sampling date (*p* = 0.3837) or host plants (*p* = 0.0589), although the latter effect did approach significance. The model also suggests that the interaction between sampling date and host plant was important (*p* = 0.0430), which is evidence that the magnitude or direction of host plant effects depended on the date that the sample was collected (Fig. 1B).

In the 2007 growing season, incidence of BLTVA in leafhoppers was considerably lower than in the two previous years, with an average of 9.2 and 5.6% infection rate in leafhoppers collected in potatoes and weeds, respectively (Fig. 1C). Statistical analysis indicated that there were no significant effects of sampling date (*p* = 0.0699), host plant (*p* = 0.1755), or the interaction between the two variables (*p* = 0.5632). Results of chi-square tests performed for each sampling date separately, testing whether BLTVA infection rates in leafhoppers were different between weed-vs. potato- collected insects within a year, showed no consistent, significant differences. Year effects were explored by first collapsing data across sampling dates within each year, and comparing years using categorical analysis. Results indicated that years differed significantly in overall BLTVA infection rates in beet leafhoppers (*p* < 0.0001). Using the data collapsed across sampling dates, there was no evidence of host effects in 2005 (*p* = 0.1773) and 2007 (*p* = 0.1767) but host effect was marginally significant for 2006 (*p* = 0.0621). However, there was evidence of an interaction between year and host plant effects (*p* = 0.0489), suggesting that direction or magnitude of host effects differed year-to-year.

During the leafhopper overwintering survey, beet leafhopper females were found and collected on overwintering weeds in several locations near potato fields throughout Yakima Valley and Columbia Basin, from Hermiston/Umatilla area in Oregon to north of Moses Lake in Washington. PCR results revealed that 29.6% of beet leafhoppers collected and tested for BLTVA phytoplasma carried the pathogen.

## Discussion

Leafhopper-transmitted phytoplasma diseases can be very damaging if not well-managed (Ploaie 1981; Lee et al. 2000). Pest management strategies for the control of phytoplasma diseases are currently directed at leafhoppers and other vectors of these plant pathogens. In addition to sampling for leafhopper abundance, it is also desirable to determine the proportions of leafhoppers that are infected with the phytoplasmas in order to effectively manage diseases caused by these plant pathogens. For example, in central United States, the aster yellows disease, which is caused by the aster yellows phytoplasma and mainly transmitted by *Macrosteles* species, causes serious damage to several vegetable crops. The aster yellows indices, based on both insect numbers and disease incidence, were developed in the Midwest and are used as action thresholds to initiate control measures before the pathogen is widely transmitted to susceptible crops (Mahr et al. 1993; Foster and Flood 1995; Capinera 2001). Control of leafhoppers using insecticides is the most effective management approach available to growers to prevent the spread of aster yellows disease (Mahr et al. 1993). To provide the most effective control, timing of insecticide applications is correlated with both leafhopper numbers and infectivity. In Wisconsin, recommendations concerning the development and severity of aster yellows are normally determined using a statewide average of leafhopper infectivity provided as a pest management service for growers (Mahr et al. 1993). The average infectivity of leafhoppers is determined from samples collected from the initial migrating leafhopper populations. Historically, leafhoppers were collected before they entered an area and given aster plants as food. The plants were then monitored for disease symptoms to estimate the insect percent infectivity and determine the number of leafhoppers that can be tolerated on a specific crop for any infectivity level. Two percent infectivity in aster leafhoppers is commonly used as baseline for action threshold development in the Midwest (Mahr et al. 1993; Foster and Flood 1995; Capinera 2001). This method can be laborious and lengthy. Recently, however, significant progress in fast detection of phytoplasmas has been made through the application of molecular biological techniques, and PCR has become the method of choice to quickly determine infectivity in leafhoppers and plants (Goodwin et al. 1999; Crosslin et al. 2006).

The beet leafhopper is a serious pest in the western United States because it transmits plant pathogens, including beet curly top virus, *Spiroplasma citri*, and BLTVA phytoplasma, to several vegetable, ornamental, and horticultural crops (Thomas and Martin 1971; Golino et al. 1989; Shaw et al. 1990, 1993; Schultz and Shaw 1991; Capinera 2001, Crosslin et al. 2005; Munyaneza and Upton 2005; Munyaneza et al. 2006a, 2008). Phytoplasma diseases have become increasingly important in vegetable crops of the Pacific Northwest. Recently, serious outbreaks of potato purple top have occurred in the Columbia Basin of Washington and Oregon and caused significant yield losses and a reduction in tuber processing quality. The beet leafhopper has been identified as the insect vectoring the BLTVA phytoplasma that causes this disease in this important potato-growing region of the United States (Munyaneza 2005; Munyaneza et al. 2007, 2008). This is a different vector than for the aster yellows phytoplasma associated with potato purple top in north central United States (Banttari et al. 1993) and Mexico (Leyva-López et al. 2002). Effective management of this potato disease in the Pacific Northwest requires knowledge of the biology and ecology of the beet leafhopper, including incidence of BLTVA phytoplasma in beet leafhoppers found in and near potato fields.

The central Columbia River area of Washington and Oregon has been reported as one of the overwintering and breeding sites of the beet leafhopper in western United States (Hills 1937; Cook 1967). However, information on the BLTVA infectivity of local beet leafhopper populations in this potato-producing region is lacking. During the present study, it was determined that the beet leafhopper overwinters on weeds near potato fields throughout the Columbia Basin and Yakima Valley. Further BLTVA testing revealed that 29.6% of overwintered beet leafhopper females carried the phytoplasma. Contrary to previous beliefs, this plant pathogen apparently does not necessarily need overwintering weeds to survive the winter, which then suggests that leafhoppers might pass this phytoplasma from one season to the next.

The beet leafhopper is present in the Columbia Basin and Yakima Valley potatoes throughout the growing season. The seasonal occurrence and abundance of this insect pest in this region, including the sites surveyed during the present study, were recently described by Munyaneza et al. (2008). Results from the present study also indicated that BLTVA-infected beet leafhoppers were found in both potatoes and nearby weedy habitats throughout the growing season. Testing of beet leafhoppers by PCR showed that a large proportion of beet leafhoppers invading potatoes were infected with the phytoplasma, with an average of 20.8, 34.8, and 9.2% in 2005, 2006, and 2007, respectively. These phytoplasma incidence levels in leafhoppers are particularly high compared to the 2% infectivity that is used to calculate aster yellows indices to initiate control measures of potato purple top disease in the Midwest. Results further indicated that BLTVA infection rate in the leafhoppers was variable overtime and that host plant effects (weeds vs. potato) were not evident or consistent date-to-date (Fig. 1). Moreover, year of study differed significantly in overall BLTVA infection rates in the insects, and study results suggest that the direction or magnitude of host plant effects differs year-to year. These observations suggest that incidence of BLTVA in leafhoppers from weedy habitats near potato fields may be used to relatively estimate the proportion of infective beet leafhoppers invading potatoes. This information may also be used in making decisions regarding whether to initiate control measures targeted against the beet leafhopper to reduce BLTVA incidence in potatoes.

The presence of BLTVA-infected beet leafhoppers in potatoes throughout the growing season threatens the crop by exposing potato plants to potential phytoplasma infection anytime during the season. However, to date, Columbia Basin potato growers have managed to keep the purple top disease under manageable levels by applying insecticides against the beet leafhopper early in the season (Munyaneza et al. 2006b). These observations suggest that potato plant growth stage may play an important role in susceptibility of potato to BLTVA phytoplasma. Studies to investigate this assertion are now underway, and preliminary observations indicate that, in fact, older potato plants seem less susceptible to and/or are more tolerant of the BLTVA phytoplasma (Munyaneza et al. 2010).

In summary, beet leafhoppers are found in both potatoes and nearby weeds throughout the potato-growing region of the Columbia Basin and Yakima Valley. Large proportions of these leafhoppers are infected with BLTVA phytoplasma and could infect potato plants throughout the growing season. Furthermore, a large proportion of overwintering beet leafhoppers found near potato fields carry BLTVA phytoplasma and might pass on this plant pathogen from one season to the next. Effective management strategies for phytoplasma diseases in Pacific Northwest potatoes must take into consideration BLTVA infectivity of beet leafhopper local populations invading potato fields. To provide the most effective control, timing of insecticide applications should be correlated with both beet leafhopper densities and phytoplasma infectivity. Given the high phytoplasma infectivity in beet leafhoppers in the surveyed areas compared to the 2% infectivity used in the Midwest to initiate leafhopper control, further studies are needed to accurately determine treatment thresholds for the beet leafhopper in the Pacific Northwest. Information from this study will help formulate action thresholds for beet leafhopper control to reduce damages caused by BLTVA phytoplasma to potato and other vegetable crops in affected areas. Use of action thresholds also could help reduce environmental hazards and adverse impact on non-target species caused by excessive and unnecessary pesticide applications. Moreover, manipulation of weedy habitats in the vicinity of potato fields may help reduce incidence of potato purple top disease.
